# Pay-for-performance and continuity of care synergistically reduced amputation of lower extremity in patients with diabetes: a population-based cohort study

**DOI:** 10.1186/s12913-022-08075-2

**Published:** 2022-06-04

**Authors:** Yu-Ching Chen, Yi-Han Liao, Li-Jung Elizabeth Ku, Jung-Der Wang

**Affiliations:** 1 Department of Public Health, College of Medicine, National Cheng Kung University, No.1, University Road, Tainan City, 701 Tainan, Taiwan; 2 Department of Healthcare Administration and Medical Informatics, Kaohsiung Medical University, Kaohsiung, Taiwan; 3 Department of Occupational and Environmental Medicine, National Cheng Kung University Hospital, College of Medicine, National Cheng Kung University, Tainan, Taiwan

**Keywords:** Diabetes, Pay-for-performance, Continuity of care, Lower extremity amputations

## Abstract

**Background:**

Diabetic foot is a common and costly complication of diabetes. No existing study has looked at the effect of continuity of care on amputations of diabetes (DM) patients while considering pay-for-performance (P4P) participation. We investigated the impact of the P4P program and the continuity of care index (COCI) on the incidence of lower extremity amputations (LEA) among diabetics in Taiwan.

**Methods:**

This was a population-based cohort study using insurance claims data from 1997 to 2013. We selected 15,650 DM patients in the P4P program along with age- and sex-matched non-P4P participants at a 1:4 ratio. Time-weighted average (TWA) of the COCI was calculated and included in the time-dependent Cox proportional hazard models to examine the impact of P4P and COCI on the risk of LEA, while controlling for individual and area level characteristics.

**Results:**

During four-year follow-up, 1816 subjects experienced LEA. The cumulative LEA hazard rate of the P4P group (*n* = 153) was significantly lower than that of the non-P4P group (*n* = 1663) (hazard ratio = 0.37, 95% CI = 0.31–0.43, *p* < 0.0001, by log-rank test). In the time-dependent Cox proportional hazard model, the adjusted hazard ratios (aHR) for the P4P group was 0.35, (*p* < 0.0001). With the low COCI (< 0.360) group as the reference, the aHR of LEA was 0.49 (*p* < 0.0001) for the middle COCI group, (*p* < 0.0001), and the aHR of LEA for the high COCI (≥0.643) group was 0.23 (*p* < 0.0001).

**Conclusions:**

Participating in the P4P program and increasing COCI might reduce the risk of amputation for DM patients, independently and synergistically.

**Supplementary Information:**

The online version contains supplementary material available at 10.1186/s12913-022-08075-2.

## Introduction

Twenty-five percent of diabetics with neuropathy and peripheral vascular disease will develop foot ulcers annually, [1] and up to 20% of these patients require hospitalization [2]. Of diabetes foot ulcers that do not heal, 5–24% of them will finally result in limb amputation within a period of 6–18 months after the first evaluation [3]. Foot ulcer and lower extremity vascular disease are related to a higher risk of death in diabetic patients [4]. In the United States, non-traumatic lower extremity amputations (LEA) rebounded by 50% between 2009 and 2015, driven partly by a significant 29% increase in major LEAs. On the other hand, major amputations significantly decreased with a concomitant increase in vascular interventions for patients with diabetes in Taiwan during 2007–2014 [5], and a similar trend was reported in Korea during 2011–2016 [6]. South Korea and Taiwan began implementing schemes of universal national health insurance (NHI) in 1989 and 1995, respectively [7], which features freedom from gatekeepers in seeing physician visits and easy access to vascular intervention. The diabetes care pay-for-performance (P4P) program in Taiwan was launched in a pilot trial in 1996, and then implemented nationally in 2001 [8]. Previous studies showed that patients in the diabetes P4P program had better clinical outcomes (e.g. HbA1c) [9], lower risk of LEA [10], and lower diabetes-related mortality [11]. A previous study that examined the impact of the P4P program on LEA reported that patients with diabetes (DM) who did not participate in the P4P programs had a 3.46-fold higher risk of amputation compared with P4P participants in Taiwan [10]. However, that study did not consider another important factor, namely the continuity of care (COC) in diabetes care and its association with amputations.

COC in diabetes care refers to the continuity of care a patient receives across different providers for diabetes-related outpatient visits [12, 13]. Better COC was associated with reduced healthcare expense in DM [14]; improving the COC for newly-diagnosed type 2 DM patients resulted in higher medication adherence [15, 16], lower rate of hospitalization and emergency department visits [15], and avoidable hospitalizations [17]. Besides improved patient satisfaction [18], increased COC by doctors is associated with lower mortality rates in several diseases and settings [19], including diabetes [20]. In a study that examined physician continuity, P4P program, and their association with survival among DM patients, higher physician continuity and P4P participation both had a significant independent effect on increasing survival [19]. DM patients who were also P4P participants were more likely to have better continuity of care with the same physician, and thus higher treatment quality and better survival [19, 21]. Assuming that LEA is one of important “process of care quality” measures in ambulatory settings, one would hypothesize that COC should also have a positive effect on lowering LEA of DM patients. But to our knowledge, no previous study has looked at the effect of physician continuity on amputations of DM patients. Therefore, this study aimed to simultaneously consider the independent and combined impacts of the P4P program and COC on the incidence of LEA, and our hypothesis is that the P4P and COC will have a synergistic effect in reducing amputations of lower extremity in DM patients.

## Material and method

### Data source

This was a population-based cohort study using the Taiwan National Health Insurance Research Database from 1997 to 2013. The clinical data of these patients were obtained from the following databases “Ambulatory Care Expenditures by Visits”, “Inpatient Expenditures by Admissions”, and “Healthcare Utilization Database (HUD)” [22]. The outpatient/inpatient claims used to capture the medical information included diagnostic and procedure codes, date of clinical visits, and personal identification numbers (PIDs) for both patients and physicians.

Access to the above-mentioned claims data was approved by the Health and Welfare Data Science Center (HWDC) of the Ministry of Health and Welfare. We conducted all the data collection and statistical analyses on-site at the HWDC to ensure data security. This study was approved by the Institutional Review Board of National Cheng Kung University (IRB No. AER-104-071).

### Measures

#### Pay-for-performance program (P4P)

Based on the “Ambulatory Care and Expenditures by Visits” file in 2010, patients with a reimbursement code of ‘P1401C’, ‘P1407C’, ‘P1402C’, ‘P1408C’, ‘P1403C’, ‘P1409C’, ‘P1410C’ or ‘P1411C’in their physician’s order were judged to have been enrolled into the P4P program. Code of P1407C means first enrollment into P4P program at a health care facility.

#### Continuity of care index (COCI)

There are several commonly used indicators for measuring continuity of care, including density-type usual provider continuity (UPC) index, continuity of care index (COCI), and temporality-type index (SECON index) [23]. Due to the absence of required referral arrangements and the high average annual number of physician visits in Taiwan, we chose the COCI to evaluate the continuity of care for patients because it is independent from the number of physician visits [7, 24]. In this study, all outpatient visits to Western medicine departments with diabetes-related diagnoses were extracted from the study data if ICD-9-CM was 250 or A-code was A181, and we calculated the COCI score based on diabetes-related visits with the formula proposed by Bice [25]. We believed that the diabetes-specific COCI was more sensitive for detecting the association between continuity of care and healthcare utilization for diabetic patients.

We divided the COCI scores into 3 subgroups based on the tertiles of the distribution for analysis: low (< 0.360), middle (0.360–0.643), and high (> 0.643). Moreover, the time-weighted average of continuity of care was calculated for each sample person each year to represent the cumulative effects of such activities up to the preceding month before amputation of lower extremity or being censored. Below is an example of the COCI calculation for a patient who had an LEA on July, 1, 2013:

The time-weighted average COCI formula:

**Table:** 

Year	Calculation for a patient who had an LEA on July, 1, 2013	Calculation for a control without LEA
2010	COCI_2010_	COCI_2010_
2011	(COCI_2010_+ COCI_2011_)/2	(COCI_2010_+ COCI_2011_)/2
2012	(COCI_2010_ + COCI_2011_+ COCI_2012_)/3	(COCI_2010_ + COCI_2011_+ COCI_2012_)/3
2013	(COCI_2010_+ COCI_2011_+ COCI_2012_+ COCI_2013_ × 0.5)/3.5	(COCI_2010_+ COCI_2011_+ COCI_2012_+ COCI_2013_)/4

#### Lower limb amputation (LEA) rate

From the claims data of the “Inpatient Expenditures by Admissions” files in 2010 to 2013, those patients with International Classification of Diseases, Ninth Revision, *Procedure* Coding System (ICD-9-PCS) codes initiating with 84.1, and 84.10–84.18 were identified. LEA that each diabetic underwent during those 4 yrs was identified as an event because those diabetics had been diagnosed as early as 1997

.

#### Other covariates

Covariates analyzed in this study included gender, age, first year of diabetes diagnosis, Charlson comorbidity index score (CCI), Diabetes Severity Complications Index (DCSI), Catastrophic disabling disease (CDD), level of urbanization, monthly salary/wage, level of health care facility (Table 1). Age was divided into three groups, 15–55, 56–69 and ≥ 70 years, respectively. First year of diabetes diagnosis was distributed according to calendar year. The general medical status at baseline was assessed using a modified version of the CCI, which was the sum for 19 comorbid conditions [26]. DCSI included the following 7 categories of complications: cardiovascular, nephropathy, retinopathy, peripheral vascular, neuropathy, cerebrovascular, and metabolic complications [27]. Compared with a simple count of complications, the DCSI performed slightly better and appears to be a useful tool for predicting mortality and risk of hospitalization [27]. Certificate of CDD is issued when a patient is diagnosed with one of 30 categories of catastrophic diseases [28]. Four categories of CDD are listed as follows: malignant neoplasms requiring long-term therapy; chronic kidney disease, stage V or dialysis; rheumatologic disorders requiring life-long therapy; mental disorders including dementia, schizophrenia, affective disorders, and others. Monthly salary/wage served as a proxy indicator of individual income [29] and was classified into one of 3 categories: fixed premium and dependent, less than NTD 20,000 monthly, and NTD 20,000 or more monthly (average exchange rate New Taiwan Dollar 31.3 = USD 1.0 in 2010). The fixed premium group included those receiving social welfare supports such as veterans, low-income individuals and the indigenous people of Taiwan. The dependent insurance premium group comprised spouse and dependents who did not have a job or income.

**Table 1:** Demographic of study cohort by pay-for-performance (P4P) and exact matching and propensity

	Before “exact and PS matching”			After “exact and PS matching”		
Characteristics	P4P (%)	Non-P4P (%)	***P***	P4P (%)	Non-P4P (%)	***P***
Gender			< 0.0001			1.0
Total	18,447	140,938		15,650	62,600	
Female	9280 (50.31%)	65,827 (46.71%)		7442 (47.55%)	29,768 (47.55%)	
Male	9167 (49.69%)	75,111 (53.29%)		8208 (52.45%)	32,832 (52.45%)	
Age group, (y)			< 0.0001			0.99
18–55	3651 (19.79%)	25,455 (18.06%)		2422 (15.48%)	9666 (15.44%)	
56–69	7618 (41.3%)	49,309 (34.99%)		6535 (41.76%)	26,155 (41.78%)	
≥70	7178 (38.91%)	66,174 (46.95%)		6693 (42.77%)	26,779 (42.78%)	
Age, y, mean ± SD	65.11 ± 11.88	67.35 ± 12.46	< 0.0001	66.71 ± 10.41	66.71 ± 10.41	0.99
Duration of diabetes (y)	8.55 ± 3.61	7.48 ± 4.23	< 0.0001	8.69 ± 8.63	8.69 ± 8.66	0.88
CCI			< 0.0001			0.39
0	9212 (49.94%)	61,011 (43.29%)		8178 (52.26%)	32,775 (52.36%)	
1	2903 (15.74%)	21,554 (15.29%)		2473 (15.8%)	9987 (15.95%)	
2	2429 (13.17%)	18,201 (12.91%)		1985 (12.68%)	7568 (12.09%)	
3	1526 (8.27%)	13,073 (9.28%)		1194 (7.63%)	4772 (7.62%)	
4	899 (4.87%)	9176 (6.51%)		699 (4.47%)	2915 (4.66%)	
≥5	1478 (8.01%)	17,923 (12.72%)		1121 (7.16%)	4583 (7.32%)	
CCI, mean ± SD	1.42 ± 2.11	1.84 ± 2.44	< 0.0001	1.31 ± 2.02	1.32 ± 2.02	0.75
DCSI			< 0.0001			0.40
0	13,296 (72.08%)	86,765 (61.56%)		11,261 (71.96%)	44,910 (71.74%)	
1–2	3506 (19.01%)	29,820 (21.16%)		3032 (19.37%)	12,046 (19.24%)	
≥3	1645 (8.92%)	24,353 (17.28%)		1357 (8.67%)	5644 (9.02%)	
DCSI, mean ± SD	0.69 ± 1.55	1.23 ± 2.29	< 0.0001	0.64 ± 1.37	0.66 ± 1.38	0.25
CDD	3352 (18.17%)	25,832 (18.33%)	0.603	2220 (14.19%)	8932 (14.27%)	0.79
Residence			< 0.0001			0.60
Rural	5430 (29.44%)	39,085 (27.73%)		4428 (28.29%)	17,581 (28.08%)	
Urban	13,017 (70.56%)	10,1853 (72.27%)		11,222 (71.71%)	45,019 (71.92%)	
Monthly salary/wage†			< 0.0001			0.02
FP and dependent	7754 (42.03%)	59,943 (42.53%)		6884 (43.99%)	28,289 (45.19%)	
Less than NTD† 20,000	3606 (19.55%)	33,919 (24.07%)		3215 (20.54%)	12,568 (20.08%)	
NTD 20,000 or more	7087 (38.42%)	47,076 (33.4%)		5551 (35.47%)	21,743 (34.73%)	
Health care facility level			< 0.0001			0.49
Medical center	4775 (25.88%)	41,426 (29.39%)		4316 (27.58%)	17,349 (27.71%)	
Regional hospital	5676 (30.77%)	37,107 (26.33%)		4108 (26.25%)	16,501 (26.36%)	
District hospital	3647 (19.77%)	27,554 (19.55%)		3242 (20.72%)	12,626 (20.17%)	
Community clinic	4349 (23.58%)	34,851 (24.73%)		3984 (25.46%)	16,124 (25.76%)	

*P* = *p*-value

*P4P* pay-for-performance, *SD* Standard deviation, *CCI* Charlson comorbidity index, *DSCI *diabetes severity comorbidity index, *CDD *catastrophic disabling disease, *FP *fixed premium

† 1USD = 31.3 New Taiwan Dollars (NTD) in 2010

### Participants

Patients with diabetes in 2010 were selected from the Taiwan National Health Insurance (NHI) database. The regularity of visits and the survival status of these patients were followed for 4 yrs until 2013. Diabetes patients (ICD-9-CM code 250 or A181 in A-code) with at least 3 outpatient diagnoses or one inpatient admission in 2010 were included. The validation for this definition of diabetes showed a 96.9% sensitivity and 93.9% positive predictive value in a study using a questionnaire assessment of diabetes patients from NHIRD [30]. The exclusion criteria were as follows: (1) equal to or less than two outpatient diabetes diagnosis in 2010; (2) age < 18 years because our analysis focused on type 2 diabetes patients.

**Fig. 1:**
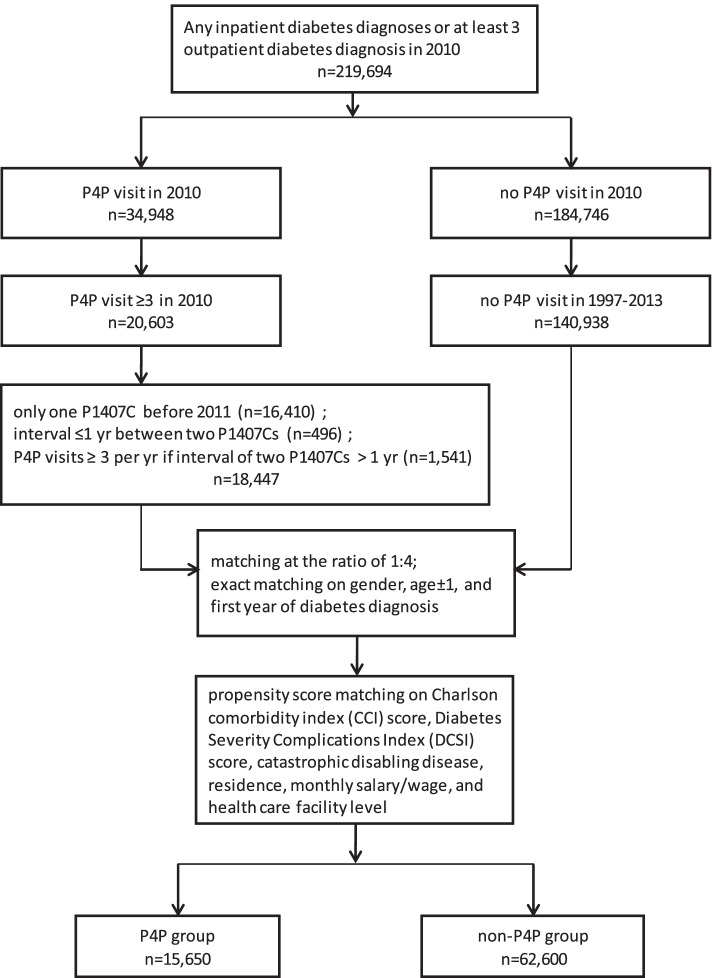
Flow diagram of study cohort

Figure 1 shows that diabetics with P4P visits ≥3 times in 2010 were screened to include (a) those with only one ‘P1407C’ before 2011 (*n* = 16,410); (b) those with interval ≤ 1 year between two ‘P1407Cs’ before 2011 (*n* = 496), and (c) those with P4P visits ≥3 times per year if interval between two ‘P1407C’ was more than 1 year before 2011 (*n* = 1541). The sum of these three subgroups is 18,472. For non-P4P group, diabetics without P4P visit from 1997 to 2013 were screened and matched. In order to mitigate confounding, exact matching on gender, age ± 1, and first year of diabetes diagnosis, and propensity score matching on Charlson comorbidity index score, diabetes severity complication index score, catastrophic disabling disease, residence, monthly salary/wage, and health care facility level at the ratio of 1:4 was done. For every intervention group subject, four controls were matched for the non-P4P group. The final sample size obtained was 15,650 subjects in the P4P group and 62,600 subjects in the non-P4P group.

### Statistical analysis

Descriptive statistics were calculated and the χ2 test was used for categorical covariates and independent t-test for continuous covariates to compare whether significant differences existed between P4P participants and non-participants at baseline. Cumulative LEA-free rate was assessed by Kaplan-Meier analysis. Three Cox proportional hazard models, including two time-dependent ones, were constructed to determine the impact of physicians’ continuity of care on the risk of LEA for diabetes patients after adjusting for P4P program participation, gender, age, duration of diabetes, CCI, DCSI, CDD, residence, monthly salary/wage and health care facility level (Table 2). In model A, cumulatively estimated for consecutive years beginning with 2010 during 2010–2013, time-weighted average COCIs, divided into three tertiles, were time-dependent and varied yearly from 2010 to 2013. In model B, P4P and four-year average COCI were integrated into a variable, where six subgroups were produced. For sensitivity analysis, we treated the time-weighted average COCIs as a time-dependent continuous variable in model C. Subgroup analyses for all covariates and interaction with P4P program for the main model A were conducted and listed in Table 3. SAS version 9.4 (SAS Institute, Cary, North Carolina) was used to process and analyze the data.\

**Table 2:** Adjusted hazard ratios by Cox proportional hazard model for different risk factors of LEA

Covariate	Adjusted HR (95%CI)	Adjusted HR (95% CI)	Adjusted HR (95% CI)
	Model A^a^	Model B^b^	Model C^c^
**P4P** (ref.: non-P4P)	0.35 (0.29-0.41)*		0.37 (0.30-0.44)*
**COCI**			0.08 (0.06-0.10)*
Low COCI (reference)			
Middle COCI	0.49 (0.43-0.55)*		
High COCI	0.23 (0.21-0.27)*		
**P4P & COCI**			
non-P4P, low COCI(reference)			
non-P4P, middle COCI	0.68 (0.61-0.76)*		
non-P4P, high COCI	0.26 (0.22-0.31)*		
P4P, low COCI	0.53 (0.44-0.67)*		
P4P, middle COCI	0.30 (0.23-0.38)*		
P4P, high COCI	0.06 (0.04-0.10)*		
**Gender** (ref.: female)			
Male	1.16 (1.04-1.29)ǂ	1.09 (0.99-1.20)	1.15 (1.02-1.29)+
**Age** (ref.: 18< yr ≤ 55)			
56 ≤ yr ≤ 69	0.85 (0.73-1.00)+	0.81 (0.70-0.94)ǂ	0.89 (0.75-1.06)
yr ≥ 70	0.71 (0.60-0.83)*	0.59 (0.51-0.69)*	0.73 (0.61-0.87)*
**Diabetes** **duration **(ref.: <5 yr)			
5 ≤ duration <10	2.06 (1.54-2.76)*	2.29 (1.76-2.98)*	2.09 (1.52-2.87)*
duration≥ 10	3.91 (2.93-5.20)*	4.35 (3.36-5.63)*	3.90 (2.85-5.32)*
**CCI score** (ref: score=0)			
1-2	0.55 (0.47-0.64)*	0.56 (0.48-0.64)*	0.55 (0.47-0.66)*
≥ 3	0.28 (0.21-0.39)*	0.31 (0.24-0.41)*	0.30 (0.21-0.41)*
**DSCI score** (ref: score=0)			
1-2	1.07 (0.89-1.29)	1.05 (0.89-1.24)	1.11 (0.91-1.35)
≥ 3	1.77 (1.28-2.45)*	1.63 (1.21-2.19)ǂ	1.89 (1.34-2.65)*
**CDD** (ref: No)			
Yes	0.72 (0.56-0.93)+	0.82 (0.65-1.05)	0.77 (0.58-1.01)
**Residence** (ref.: Rural)			
Urban	0.82 (0.73-0.92)*	0.81 (0.73-0.91)*	0.83 (0.73-0.94)ǂ
**Monthly salary/wage** (ref.: FP and dependent)			
< NTD 20,000	0.96 (0.83-1.10)	0.96 (0.84-1.09)	1.00 (0.86-1.15)
≥ NTD 20,000	0.85 (0.75-0.97)+	0.86 (0.76-0.96)ǂ	0.91 (0.80-1.04)
**Health care facility level** (ref.: Medical center)			
Regional hospital	1.14 (1.00-1.31)	1.11 (0.98-1.26)	1.13 (0.98-1.31)
District hospital	1.06 (0.92-1.23)	1.02 (0.89-1.17)	0.99 (0.83-1.16)
Community clinic	0.87 (0.75-1.01)	0.86 (0.75-0.99) +	0.89 (0.75-1.05)
**Akaike information criterion**	30,787	36,804	30,699
**Schwarz-Bayesian criterion**	30,888	36,918	30,794

*p<0.001; ǂ *p*<0.01; + *p*<0.05

a: Categorical time-dependent time-weighted average COCI, b: Stratification of average time weighted-average COCI by P4P, c: Continuous time-weighted average COCI, ref: reference; TWA= time-weighted average; P4P= pay for performance; COCI: continuity of care index; Int=Intermediate COCI; CCI= Charlson

**Table 3:**
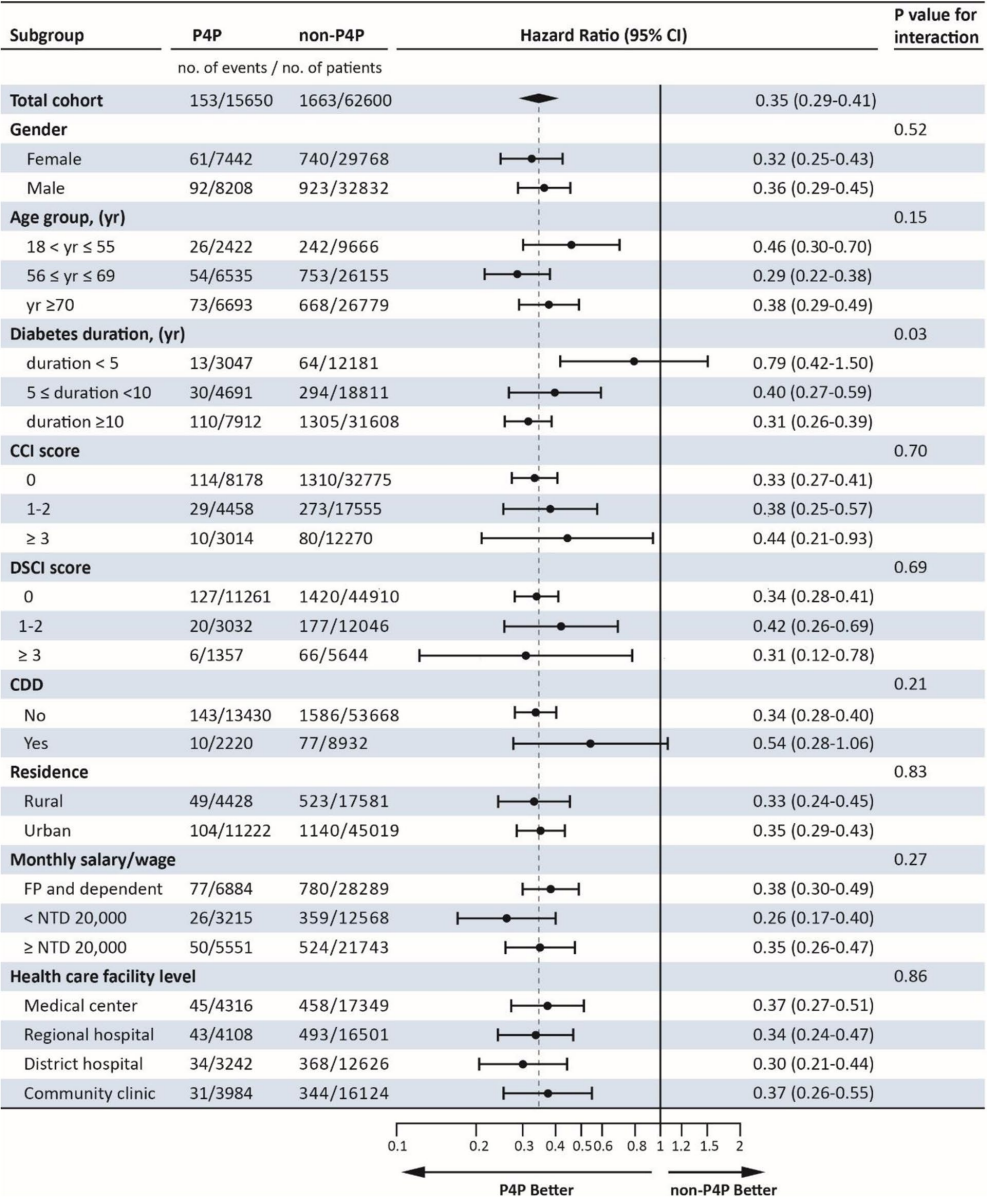
Subgroup analysis of risk factors for the hazard of lower extremity amputation by P4P status.

*P4P* = Pay for performance, *CCI* = Charlson comorbidity index, *DCSI* = Diabetes Complications Severity index, *CDD* = Catastrophic disabling disease, *FP* fixed premium

## Result

Table 1 compares the baseline characteristics of the enrolled subjects with diabetes, including 15,650 under P4P care and 62,600 non-participants at the ratio of 1:4. The average age was 66.7 years old for both groups, 41.8% were 56–69 years old, and nearly 48% were women. Duration of diabetes was 8.69 years. At baseline, after exact, and propensity score, matching, no significant difference existed between P4P group and non-P4P group in all covariates except monthly salary/wage (*p* = 0.02). Supplementary eTable 1 in the Appendix presents results on the exact matching of first year of diabetes diagnosis.

**Fig. 2:**
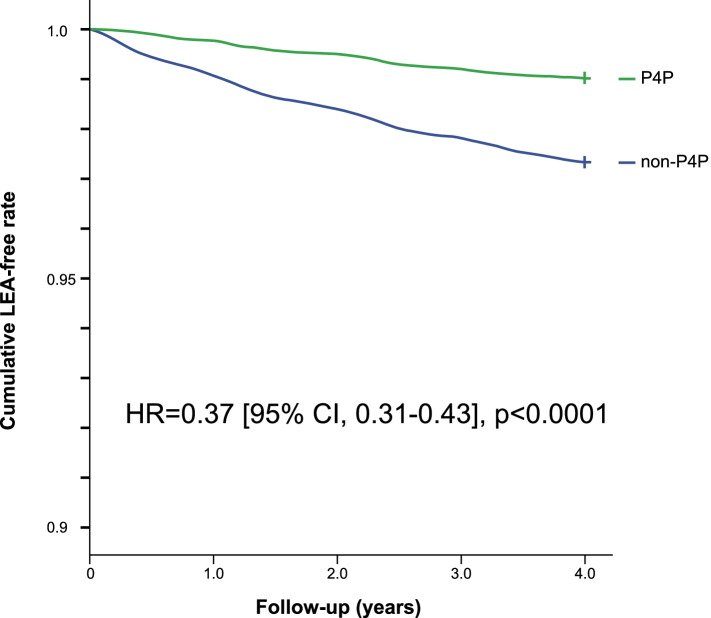
Kaplan-Meier LEA-free curve by pay-for-performance in Taiwan. During four-year follow-up, the cumulative LEA-free rate of the P4P group was significantly higher than that of the non-P4P group (hazard ratio, 0.37 [95% CI, 0.31–0.43], *p* < 0.0001, by log-rank test)

In Fig. 2, Kaplan–Meier analysis showed that during four-year follow-up, the cumulative LEA-free rate of the P4P group was significantly higher than that of non-P4P group (hazard ratio = 0.37 [95% confidence interval, 0.31–0.43], *p* < 0.0001, by log-rank test).

Table 2 summarizes the results of three models of Cox proportional hazard, including two time-dependent, time-weighted average COCI models, with the COCI groups as a categorical variable in model A, but as a continuous variable in model C. They were constructed to adjust the other nine confounders listed in Table 1. In the main model A, the aHR of occurrence of LEA for P4P participants was 0.35 (*p* < 0.0001) compared with non-participants. With the low COCI (< 0.360) group as the reference, the aHR of LEA was 0.49 (*p* < 0.0001) for the middle COCI group, *p* < 0.0001, and the aHR of LEA for the high COCI (≥0.643) group was 0.23 (*p* < 0.0001). Compared to women, men had a higher aHR of 1.16 for LEA (*p* = 0.006). A dose-response relationship was observed for the association between diabetes duration and LEA and association between the DCSI and LEA. Compared to patients with diabetes duration less than 5 years, those with duration 5–10 years and ≥ 10 years were shown to be 2.06 (*p* < 0.0001) and 3.91-fold (p < 0.0001) more likely to undergo amputation, respectively. Compared to those DCSI score = 0, patients with a score of 2 and ≥ 3 were found to be 1.07 (*p* = 0.46) and 1.77 (*p* = 0.0006) times more likely to have an amputation, respectively. Those living in urban areas had a lower risk of LEA than those living in rural areas (aHR = 0.82, *p* = 0.0008). Community clinic bore a trend with lower rate of LEA (aHR = 0.87, *p* = 0.08) in comparison to medical center. In model B, with low COCI (< 0.360) subgroup of non-P4P group as the reference, the aHR of LEA was 0.68 (*p* < 0.0001) for middle COCI subgroup of non-P4P group, 0.26 (*p* < 0.0001) for high COCI (≥0.643) subgroup of non-P4P group, 0.53 (*p* < 0.0001) for low COCI subgroup of P4P group, 0.30 (p < 0.0001) for middle COCI subgroup of P4P group, and 0.06 (*p* < 0.0001) for high COCI subgroup of P4P group, respectively. In sensitivity analysis results shown in model C, the aHR of the occurrence of LEA for P4P group was 0.37 (*p* < 0.0001), compared with non-P4P group, and the aHR for COCI was as low as 0.08. Model C has the lowest Akaike information criterion (AIC) and Schwarz-Bayesian criterion (SBC), indicating improved model fit.

In Table 3, using time-dependent model A, major subgroup analyses were performed according to risk factors listed in Table 1. The benefit of P4P with respect to lower extremity amputation tended to be similar across subgroups, except in diabetes duration where *p*-value for interaction 0.03. When only time-weighted average COCI of 4-year duration, namely 2010 ~ 2013, was counted, and model A was not time-dependent, subgroup analysis for COCI was obtained; p-value for interaction was 0.02. The result was shown in supplementary eTable 2.

## Discussion

In this study, although we found that both P4P and continuity of care had a lower HR of amputations among DM patients, it does not necessarily imply that such an association is likely to be causal. However, we have the following arguments to support the hypothesis of a potential synergistic effect between P4P and continuity of care in reducing LEA: First, we used a nation-wide database of Taiwan, which includes records of the beneficiaries in the National Health Insurance, accounting for 99.8% of the total population in Taiwan. This makes the large sample size highly representative of the study cohort. Second, since we controlled for the potential confounding factors of gender, age, diabetes duration, residence, monthly salary/wage, health care facility level and major comorbidities (through Charlson comorbidity index, DCSI and catastrophic disabling disease) in the Cox model construction, the above factors cannot be used to explain the estimated aHRs between amputation and P4P or COCI. Third, we took the time-weighted average of COCI as a measurement of continuity of care and modeled it as a time-dependent variable in the Cox proportional hazard model, which represents an improvement over existing studies that used a time-invariant measure of continuity of care. Our findings also showed that estimation result using time-weighted average of COCI was robust when the measure was modeled as either a categorical or a continuous variable. While model C with the COCI as a continuous variable seemed to be the model with better model fit, we still chose the categorical model A as our preferred model because the literature has suggested that the COCI values have no inherent clinical meaning, and therefore, were usually modeled as tertiles instead of as a continuous variable [16]. Lastly, as LEA in DM patients usually result from neuropathy, trauma, and peripheral vascular disease, our findings on the possible synergistic effect of both P4P and COC corroborates previous reports which showed that both factors reduce mortality in diabetes [19], usually resulting from macro-vascular complications, such as coronary artery disease, cerebral vascular disease, etc. [31]. Namely, the pathophysiologic mechanism is plausible. Therefore, we tentatively conclude that the association may exist and deserve further attention.

The results from our study showed that the aHRs of LEA for participants in the DM P4P program was 0.35 (95% CI, 0.29–0.41) compared with non-participants. This aHR estimate had a smaller effect size than that in a previous study by Sheen at el. which found an aHR of LEA of 0.29 for DM P4P (calculated from 1/3.46 for non-P4P participants) [10]. While findings from both studies support that DM P4P has a protective effect on LEA, the following reasons might explain the differences in findings: First, our study of 4 years was only four tenths of their study duration, and the HR of amputation would decrease over time with the advent of enhanced care and new technology. In addition, we applied exact matching on gender, age and first year of diabetes diagnosis and PS matching on other covariates in our study, which may narrow the difference in LEA between the P4P and non-P4P groups, resulting in a HR closer to one. Furthermore, Sheen et al. did not investigate the impact of continuity of care on lower extremity amputation as we did. High COCI turned out to be a strong protective factor both in P4P group (adjusted HR = 0.06) and in non-P4P group (adjusted HR = 0.26) in model B of Table 2. Finally, we have included time-weighted averages of COCI in time-dependent Cox models, and all of these models showed robustness for the synergistic effects, based on two different model fit criteria.

In line with our hypothesis that physician continuity would lead to better care outcomes, our findings showed that the aHRs of LEA for patients in the middle or high COCI group was between 0.23–0.49 compared with the low COCI group. While our study seems to be the first to report the negative association between physician continuity and amputation, this result is consistent with findings from previous studies on diabetes care: Subjects that had a regular health care provider were more likely than those without to receive higher frequency of glycosylated hemoglobin (HbA1c) testing and more foot examinations (42% vs 17%) [32]. The P4P program in Taiwan mandates that a foot examination [33], which includes artery palpation, Semmes-Weinstein monofilament examination, and vibration perception [34], to be conducted at the enrollment visit, annual visit and, if indicated, interposed quarterly visits. Therefore, when DM patients are enrolled into the P4P program, they are more likely to follow a structured education program, and thereby, have higher adherence to medications [15, 16], and by screening for neuropathy which can be serious and prevent ulcerations from being noticed, the P4P program would mitigate the risk of ulcerations, severe infections, and eventually, amputations.

In addition to our main findings on the association between P4P, COCI, and LEA, results from our Cox proportional hazard model also examined other risk factors on LEA. Our study showed that males had a higher risk of LEA, and this was comparable to the preventive recommendations by the American Diabetes Association [35] and findings from another previous study [10, 36]. Compared to the group of “fixed premium or dependent”, group of “≥ NTD 20,000” had a lower risk of LEA. This finding was similar to the study by Sheen et al., as that study also showed that patients who had lower incomes had higher amputation rates [10]. The risk of LEA in those with DCSI scores ≥3 was about 1.8 times more than those without diabetic related complications. The study by Sheen et al. also showed that the higher the number of diabetic-related complications, the higher the risk of receiving LEA [10]. The authors found that when diabetes patients had other complications, the prognosis was seriously bad since foot lesions could not be controlled and amputation was required [10].

There are some limitations in this study that must be acknowledged: First, this is not a randomized control trial; therefore, some unmeasured confounders cannot be ruled out despite exact, and propensity score, matching. Second, limiting P4P enrollees to those with P4P visits ≥3 times per year, this study cannot be generalized to all P4P enrollees. Third, since the calculation of the COCI, by definition, excluded patients with less than 3 physician visits a year, that exclusion criteria may also limit the generalizability of our findings to all DM patients. Fourth, we used the NHI claims data which does not contain the risk factors of diabetic foot ulcer, including smoking, obesity, low-density lipoprotein cholesterol and ankle-brachial index [37, 38]. Fifth, the claims data also did not include the severities of peripheral artery disease, and its association with vascular interventions and/or amputations could not be explored. However, as the smoking rate for females in Taiwan has generally been less than 5% in the last 3 decades, while that of males has been about 34–45% [39], we are not surprised that male DM patients showed an aHR of LEA about 1.2 times that of females and this could partially explain the effect of smoking on LEA. Moreover, other related vascular risk factors may also be partially adjusted by the inclusion of stratified DCSI in our regression models Thus, the potential confounding caused by these unmeasured risk factors may not be high.

## Conclusion

By using time-weighted average methods to calculate the COCI for diabetes-related visits, this study contributed new insight into the association between continuity of care and amputations. This large population-based cohort study concluded that the P4P program and COC might synergistically reduce the risk of LEA in DM patients. Thus, more studies, possibly including randomized control trials, would be warranted to corroborate the above findings for further improvement of diabetic care policy.

## Supplementary Information

**Additional file 1.**

## Data Availability

The data that support the findings of this study are available from the Health and Welfare Data Science Center (HWDC) of Taiwan, but restrictions apply to the availability of these data, which were applied to be used exclusively for the current study, and so are not publicly available.

## Abbreviations

DM: Diabetes
P4P: Pay-for-Performance
COC: Continuity of Care LEA Lower extremity amputations aHR adjusted Hazard Ratios

## References

[1] Lepantalo M, Apelqvist J, Setacci C, Ricco JB, de Donato G, Becker F (2011). Chapter V: diabetic foot. Eur J Vasc Endovasc Surg.

[2] Bandyk DF (2018). The diabetic foot: pathophysiology, evaluation, and treatment. Semin Vasc Surg.

[3] Alexiadou K, Doupis J (2012). Management of diabetic foot ulcers. Diabetes Ther.

[4] Boyko EJ, Ahroni JH, Smith DG, Davignon D (1996). Increased mortality associated with diabetic foot ulcer. Diabet Med.

[5] Lin CW, Armstrong DG, Lin CH, Liu PH, Hung SY, Lee SR (2019). Nationwide trends in the epidemiology of diabetic foot complications and lower-extremity amputation over an 8-year period. BMJ Open Diabetes Res Care.

[6] Kim J, Chun DI, Kim S, Yang HJ, Kim JH, Cho JH (2019). Trends in lower limb amputation in patients with diabetic foot based on vascular intervention of peripheral arterial disease in Korea: a population-based Nationwide study. J Korean Med Sci.

[7] Cheng SH, Jin HH, Yang BM, Blank RH (2018). Health expenditure growth under single-payer systems: comparing South Korea and Taiwan. Value Health Reg Issues.

[8] National Health Insurance Administration Ministry of Health and Welfare. The Pay-for-Performance Program for Diabetes under National Health Insurance in Taiwan 2012 [Available from: https://www.nhi.gov.tw/Content_List.aspx?n=95611DD9DDCAF987&topn=5FE8C9FEAE863B46.

[9] Chiu HC, Hsieh HM, Lin YC, Kuo SJ, Kao HY, Yeh SC (2016). Patient assessment of diabetes care in a pay-for-performance program. Int J Qual Health Care.

[10] Sheen YJ, Kung PT, Kuo WY, Chiu LT, Tsai WC (2018). Impact of the pay-for-performance program on lower extremity amputations in patients with diabetes in Taiwan. Medicine (Baltimore).

[11] Hsieh HM, Chiu HC, Lin YT, Shin SJ (2017). A diabetes pay-for-performance program and the competing causes of death among cancer survivors with type 2 diabetes in Taiwan. Int J Qual Health Care.

[12] Hanninen J, Takala J, Keinanen-Kiukaanniemi S (2001). Good continuity of care may improve quality of life in type 2 diabetes. Diabetes Res Clin Pract.

[13] Chen CC, Cheng SH (2016). Does pay-for-performance benefit patients with multiple chronic conditions? Evidence from a universal coverage health care system. Health Policy Plan.

[14] Chen CC, Chen SH (2011). Better continuity of care reduces costs for diabetic patients. Am J Manag Care.

[15] Chen CC, Tseng CH, Cheng SH (2013). Continuity of care, medication adherence, and health care outcomes among patients with newly diagnosed type 2 diabetes: a longitudinal analysis. Med Care.

[16] Chen CC, Cheng SH (2016). Continuity of care and changes in medication adherence among patients with newly diagnosed diabetes. Am J Manag Care.

[17] Lin W, Huang IC, Wang SL, Yang MC, Yaung CL (2010). Continuity of diabetes care is associated with avoidable hospitalizations: evidence from Taiwan's National Health Insurance scheme. Int J Qual Health Care.

[18] van Walraven C, Oake N, Jennings A, Forster AJ (2010). The association between continuity of care and outcomes: a systematic and critical review. J Eval Clin Pract.

[19] Pan CC, Kung PT, Chiu LT, Liao YP, Tsai WC (2017). Patients with diabetes in pay-for-performance programs have better physician continuity of care and survival. Am J Manag Care.

[20] Pereira Gray DJ, Sidaway-Lee K, White E, Thorne A, Evans PH. Continuity of care with doctors-a matter of life and death? A systematic review of continuity of care and mortality. BMJ Open 2018;8(6):e021161. 10.1136/bmjopen-2017-021161 PMC6042583 29959146

[21] Yen SM, Kung PT, Sheen YJ, Chiu LT, Xu XC, Tsai WC (2016). Factors related to continuing care and interruption of P4P program participation in patients with diabetes. Am J Manag Care.

[22] Li-Jung Elizabeth Ku C-CL, Chung-Yi Li. The Establishment and Application of Healthcare Utilization Database in Taiwan. J Health Sci,. 2018;5 (Supple I):11–23.

[23] Shortell SM (1976). Continuity of medical care: conceptualization and measurement. Med Care.

[24] Smedby O, Eklund G, Eriksson EA, Smedby B. Measures of continuity of care. A register-based correlation study Medical care 1986;24(6):511–518. 10.1097/00005650-198606000-00005 3713289

[25] Bice TW, Boxerman SB (1977). A quantitative measure of continuity of care. Med Care.

[26] Elixhauser A, Steiner C, Harris DR, Coffey RM (1998). Comorbidity measures for use with administrative data. Med Care.

[27] Young BA, Lin E, Von Korff M, Simon G, Ciechanowski P, Ludman EJ (2008). Diabetes complications severity index and risk of mortality, hospitalization, and healthcare utilization. Am J Manag Care.

[28] Catastrophic disabling disease. January 22nd, 2022 [in Chinese]. https://www.nhigovtw/Content_Listaspx?n=3AE7F036072F88AF. 2022.

[29] Wen CP, Tsai SP, Chung WS (2008). A 10-year experience with universal health insurance in Taiwan: measuring changes in health and health disparity. Ann Intern Med.

[30] Lin CC, Lai MS, Syu CY, Chang SC, Tseng FY (2005). Accuracy of diabetes diagnosis in health insurance claims data in Taiwan. J Formos Med Assoc.

[31] Stern JR, Wong CK, Yerovinkina M, Spindler SJ, See AS, Panjaki S (2017). A Meta-analysis of long-term mortality and associated risk factors following lower extremity amputation. Ann Vasc Surg.

[32] O'Connor PJ, Desai J, Rush WA, Cherney LM, Solberg LI, Bishop DB (1998). Is having a regular provider of diabetes care related to intensity of care and glycemic control?. J Fam Pract.

[33] Chen YC, Lee CT, Lin BJ, Chang YY, Shi HY (2016). Impact of pay-for-performance on mortality in diabetes patients in Taiwan: a population-based study. Medicine (Baltimore).

[34] Smieja M, Hunt DL, Edelman D, Etchells E, Cornuz J, Simel DL (1999). Clinical examination for the detection of protective sensation in the feet of diabetic patients. International cooperative Group for Clinical Examination Research. J Gen Intern Med.

[35] American Diabetes Association (2004). Preventive foot Care in Diabetes. Diabetes Care.

[36] Lin C, Liu J, Sun H (2020). Risk factors for lower extremity amputation in patients with diabetic foot ulcers: a meta-analysis. PLoS One.

[37] Aziz Z, Lin WK, Nather A, Huak CY. Predictive factors for lower extremity amputations in diabetic foot infections. Diabet. Foot Ankle. 2011;2. 10.3402/dfa.v2i0.7463 PMC3284283 22396824

[38] Singer AJ, Tassiopoulos A, Kirsner RS (2017). Evaluation and Management of Lower-Extremity Ulcers. N Engl J Med.

[39] Chiang CY, Chang HY (2016). A population study on the time trend of cigarette smoking, cessation, and exposure to secondhand smoking from 2001 to 2013 in Taiwan. Popul Health Metrics.

