# Correction: Pay-for-performance and continuity of care synergistically reduced amputation of lower extremity in patients with diabetes: a population-based cohort study

**DOI:** 10.1186/s12913-022-08368-6

**Published:** 2022-08-12

**Authors:** Yu-Ching Chen, Yi-Han Liao, Li-Jung Elizabeth Ku, Jung-Der Wang

**Affiliations:** 1 Department of Public Health, College of Medicine, National Cheng Kung University, No.1, University Road., 701 Tainan, Taiwan; 2 Department of Healthcare Administration and Medical Informatics, Kaohsiung Medical University, Kaohsiung, Taiwan; 3 Department of Occupational and Environmental Medicine, National Cheng Kung University Hospital, College of Medicine, National Cheng Kung University, Tainan, Taiwan

**Correction: BMC Health Serv Res 22, 748 (2022)**

**https://doi.org/10.1186/s12913-022-08075-2**

Following publication of the original article [1], some numbers under the heading **P4P & COCI in Table 2** needs to be moved from the column **Model A**^**a**^ to the column **Model B**^**b**^. This problem is caused due to a typesetting error. The correct Table 2 is given below.

**Table 2:** Adjusted hazard ratios by Cox proportional hazard model for different risk factors of LEA

Covariate	Adjusted HR (95%CI)	Adjusted HR (95% CI)	Adjusted HR (95% CI)
	Model A^**a**^	Model B^**b**^	Model C^**c**^
**P4P** (ref.: non-P4P)	0.35 (0.29-0.41)*		0.37 (0.30-0.44)*
**COCI**			0.08 (0.06-0.10)*
Low COCI (reference)			
Middle COCI	0.49 (0.43-0.55)*		
High COCI	0.23 (0.21-0.27)*		
**P4P & COCI**			
non-P4P, low COCI (reference)			
non-P4P, middle COCI		0.68 (0.61-0.76)*	
non-P4P, high COCI		0.26 (0.22-0.31)*	
P4P, low COCI		0.53 (0.44-0.67)*	
P4P, middle COCI		0.30 (0.23-0.38)*	
P4P, high COCI		0.06 (0.04-0.10)*	
**Gender** (ref.: female)			
Male	1.16 (1.04-1.29) ǂ	1.09 (0.99-1.20)	1.15 (1.02-1.29)+
**Age** (ref.: 18< yr ≤ 55)			
56 ≤ yr ≤ 69	0.85 (0.73-1.00)+	0.81 (0.70-0.94) ǂ	0.89 (0.75-1.06)
yr ≥ 70	0.71 (0.60-0.83)*	0.59 (0.51-0.69)*	0.73 (0.61-0.87)*
**Diabetes duration** (ref.: <5 yr)			
5 ≤ duration <10	2.06 (1.54-2.76)	2.29 (1.76-2.98)*	2.09 (1.52-2.87)*
duration≥ 10	3.91 (2.93-5.20)	4.35 (3.36-5.63)*	3.90 (2.85-5.32)*
**CCI score** (ref: score=0)			
1-2	0.55 (0.47-0.64)*	0.56 (0.48-0.64)*	0.55 (0.47-0.66)*
≥ 3	0.28 (0.21-0.39)*	0.31 (0.24-0.41)*	0.30 (0.21-0.41)*
**DSCI score** (ref: score=0)			
1-2	1.07 (0.89-1.29)	1.05 (0.89-1.24)	1.11 (0.91-1.35)
≥ 3	1.77 (1.28-2.45)*	1.63 (1.21-2.19) ǂ	1.89 (1.34-2.65)*
**CDD** (ref: No)			
Yes	0.72 (0.56-0.93)+	0.82 (0.65-1.05)	0.77 (0.58-1.01)
**Residence** (ref.: Rural)			
Urban	0.82 (0.73-0.92)*	0.81 (0.73-0.91)*	0.83 (0.73-0.94) ǂ
**Monthly salary/wage** (ref.: FP and dependent)			
< NTD 20,000	0.96 (0.83-1.10)	0.96 (0.84-1.09)	1.00 (0.86-1.15)
≥ NTD 20,000	0.85 (0.75-0.97)+	0.86 (0.76-0.96) ǂ	0.91 (0.80-1.04)
**Health care facility level** (ref.: Medical center)			
Regional hospital	1.14 (1.00-1.31)	1.11 (0.98-1.26)	1.13 (0.98-1.31)
District hospital	1.06 (0.92-1.23)	1.02 (0.89-1.17)	0.99 (0.83-1.16)
Community clinic	0.87 (0.75-1.01)	0.86 (0.75-0.99) +	0.89 (0.75-1.05)
**Akaike information criterion**	30,787	36,804	30,699
**Schwarz-Bayesian criterion**	30,888	36,918	30,794

*p<0.001; ǂ *p*<0.01; + *p*<0.05

^a^ Categorical time-dependent time-weighted average COCI, ^b^ Stratification of average time weightedaverage COCI by P4P, ^c^ Continuous time-weighted average COCI, *ref* Reference, *TWA* Time-weighted average, *P4P* Pay for performance, *COCI* Continuity of care index, *Int* Intermediate COCI, *CCI* Charlson comorbidity index, *DSCI* Diabetes severity comorbidity index, *CDD* Catastrophic disabling disease, *FP* Fixed premium

In calculating the cut-off points of tertiles for time-weighted average COCI 2010-2013, there was an error made in reporting so that the tertiles of the distribution of the COCI scores and thus the cutoffs should be corrected as follows:

- “low COCI (< 0.360)” revised to (<0.50);
- “middle COCI (0.360–0.643)” revised to (0.50-0.80);
- “high COCI (≥ 0.643)” revised to (≥0.80).

Numbers to be revised are shown in the following 4 sections:

**Table:** 

Page 1, Abstract, Results: With the low COCI (<0.50) group as the reference, the aHR of LEA was 0.49 (*p* < 0.0001) for the middle COCI group, (*p* < 0.0001), and the aHR of LEA for the high COCI (≥0.80) group was 0.23 (*p* < 0.0001).	
Page 3, Line 2: We divided the COCI scores into 3 subgroups based on the tertiles of the distribution for analysis: low (<0.50), middle (0.50-0.80), and high (≥0.80).	
Page 8, Left column, Line 9-12: With the low COCI (<0.50) group as the reference, the aHR of LEA was 0.49 (*p* < 0.0001) for the middle COCI group,* p* < 0.0001, and the aHR of LEA for the high COCI (≥0.80) group was 0.23 (*p* < 0.0001).	
Page 8, Right column, Line 4-8 In model B, with low COCI (<0.50) subgroup of non-P4P group as the reference, the aHR of LEA was 0.68 (*p* < 0.0001) for middle COCI subgroup of non-P4P group, 0.26 (*p* < 0.0001) for high COCI (≥0.80) …	

The original article [1] has been corrected.

## References

[1] Chen YC (2022). Pay-for-performance and continuity of care synergistically reduced amputation of lower extremity in patients with diabetes: a population-based cohort study.. BMC Health Serv Res.

