# Supplementary material for: Pay-for-performance and continuity of care synergistically reduced amputation of lower extremity in patients with diabetes: a population-based cohort study
Source: BMC Health Serv Res. 2022 Jun 4;22:748. doi: 10.1186/s12913-022-08075-2 (PMC9167521; doi:10.1186/s12913-022-08075-2)
Supplement: Supplementary file 1 — Additional file 1. [file 12913_2022_8075_MOESM1_ESM.docx]

**eTable 1.** Comparability of including first year of diabetes diagnosis as one of the criteria for exact matching

|  | Before “exact and PS matching” | | |  | After “exact and PS matching” | | |
| --- | --- | --- | --- | --- | --- | --- | --- |
| Characteristics | **P4P (%)** | **Non-P4P (%)** | ***P*** |  | **P4P (%)** | **Non-P4P (%)** | ***P*** |
| First year of diabetes diagnosis |  |  | <0.0001 |  |  |  | 1.0 |
| 1997 | 1,670 (9.05%) | 11,710 (8.31%) |  |  | 1,455 (9.3%) | 5,820 (9.3%) |  |
| 1998 | 2,669 (14.47%) | 16,034 (11.38%) |  |  | 2,396 (15.31%) | 9,584 (15.31%) |  |
| 1999 | 3,274 (17.75%) | 19,861 (14.09%) |  |  | 2,927 (18.7%) | 11,708 (18.7%) |  |
| 2000 | 2,090 (11.33%) | 14,148 (10.04%) |  |  | 1,803 (11.52%) | 7,212 (11.52%) |  |
| 2001 | 1,288 (6.98%) | 9,351 (6.63%) |  |  | 1,084 (6.93%) | 4,336 (6.93%) |  |
| 2002 | 1,178 (6.39%) | 8,163 (5.79%) |  |  | 972 (6.21%) | 3,888 (6.21%) |  |
| 2003 | 1,033 (5.6%) | 7,209 (5.12%) |  |  | 809 (5.17%) | 3,236 (5.17%) |  |
| 2004 | 1,041 (5.64%) | 7,429 (5.27%) |  |  | 824 (5.27%) | 3,296 (5.27%) |  |
| 2005 | 907 (4.92%) | 6,755 (4.79%) |  |  | 696 (4.45%) | 2,784 (4.45%) |  |
| 2006 | 819 (4.44%) | 6,874 (4.88%) |  |  | 676 (4.32%) | 2,704 (4.32%) |  |
| 2007 | 835 (4.53%) | 6,915 (4.91%) |  |  | 653 (4.17%) | 2,612 (4.17%) |  |
| 2008 | 730 (3.96%) | 7,065 (5.01%) |  |  | 582 (3.72%) | 2,328 (3.72%) |  |
| 2009 | 727 (3.94%) | 8,044 (5.71%) |  |  | 604 (3.86%) | 2,416 (3.86%) |  |
| 2010 | 186 (1.01%) | 11,380 (8.07%) |  |  | 169 (1.08%) | 676 (1.08%) |  |

**eTable 2.** Subgroup analysis of COCI for the hazard of lower extremity amputation by P4P status

**
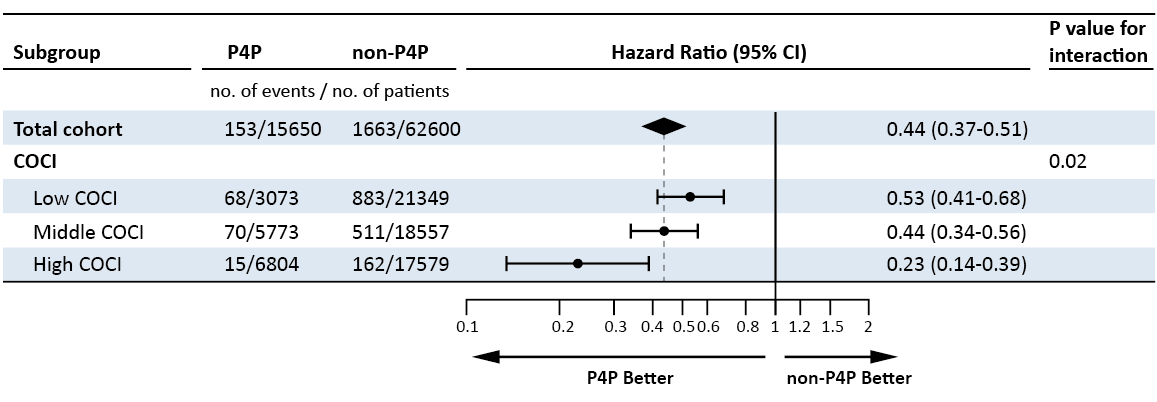
**

P4P= pay for performance; COCI= continuity of care index
